# Effects of Early Myocardial Postnatal Maturation on Tolerance to Atrial Tachycardia With Altered Loading Conditions: An *in vivo* Swine Model

**DOI:** 10.3389/fped.2020.00346

**Published:** 2020-06-25

**Authors:** Etienne Fortin-Pellerin, Nee S. Khoo, James Y. Coe, Lindsay Mills, Po-Yin Cheung, Lisa K. Hornberger

**Affiliations:** ^1^Division of Neonatology, Centre de Recherche du Centre Hospitalier Universitaire de Sherbrooke, Université de Sherbrooke, Sherbrooke, QC, Canada; ^2^Fetal and Neonatal Cardiology Program, University of Alberta, Edmonton, AB, Canada; ^3^Division of Cardiology, University of Alberta, Edmonton, AB, Canada; ^4^Departments of Pediatrics & Pharmacology, University of Alberta, Edmonton, AB, Canada

**Keywords:** neonatology, diastole, tachycardia, heart failure, maturation

## Abstract

Post-natal maturation of the myocardium starts shortly after birth and could affect how clinicians should provide hemodynamic support during this transition. Our aim was to assess the impact of post-natal maturation on tolerance to tachycardia with altered loading condition in a piglet model.

**Methods:** We report three series of experimentations. Six groups of landrace cross neonatal piglets (NP) (1–3 days) and young piglets (YP) (14–17 days) were assigned to tachycardia (NP, YP), tachycardia and hypervolemia (NPV, YPV) or tachycardia and increased afterload (NPA, YPA) groups (*n* = 7/group). Under anesthesia, a pressure catheter was placed in the left ventricle and pacing wire in the right atrium. NPV and YPV groups had 60 ml/kg of normal saline infused over 20 min. NPA and YPA had balloon sub-occlusion of the descending aorta. Heart rate was increased by 10 bpm increments to 300 bpm. Left ventricular output was measured by echocardiography.

**Results:** NP maintained left ventricular output throughout the pacing protocol but it decreased in the YP (*p* < 0.001). With volume loading both NPV and YPV maintained their output with tachycardia. Although increased afterload resulted in reduced output during tachycardia in NPA (*p* = 0.005), there was no added impact on output in YPA. Interestingly, 4 of 7 NPV had significant desaturation at 300 bpm (baseline 99.7% vs. 300 bpm 87.9%, *p* = 0.04), associated with a right to left shunt through the patent foramen ovale which resolved immediately on cessation of pacing.

**Conclusions:** Early post-natal maturation is associated with improved myocardial tolerance to increased afterload and poor tolerance of tachycardia, the latter of which may be alleviated by increasing intravascular volume. These data could translate into the development of better strategies to optimize cardiac output at these early development ages.

## Introduction

Strategies for managing neonatal shock remain controversial and the impact of rapid early post-natal development underexplored. As few randomized control trials are available to guide clinical decision making, knowledge of the maturational changes that occur in myocardial and general cardiovascular function is of great importance for optimizing bedside care. What is currently understood of the developmental changes in myocardial function from birth has largely been influenced by observations from echocardiography (1). These studies have suggested the neonatal heart has less robust diastolic function as some of the features of ventricular inflow and annular motion are similar to findings in adults with diastolic dysfunction (2). Over the first few months of life, these features evolve in a way that suggests rapid improvement in diastolic function. However, such studies have all been performed at rest. A paucity of data exists regarding the functional “reserve” of the infant heart. Furthermore, findings from cellular studies, including those examining calcium handling (3, 4) and metabolism (5), have remained difficult to translate into an anticipated clinical response of the neonatal and early infant heart to hemodynamic challenges. Moreover, the effect of maturational changes on tolerance to combined hemodynamic stressors, a common occurrence in neonatal and pediatric intensive care, has not been sufficiently explored.

Using a piglet model of rapid atrial pacing, we previously demonstrated that tolerance to tachycardia actually decreases in the first few weeks (6), a finding associated with differences in the twist pattern of the left ventricle (LV). Whether changes in preload and afterload impact the tolerance of the neonatal and young infant heart to tachycardia is not certain. It has been suggested that post-natal maturation increases the myocardial response to preload (7–10); however, animal models have provided evidence that preload reserve exists even in preterm hearts (11). Although past studies have also suggested the immature heart is afterload sensitive (9, 10), whether contractility is augmented though tachycardia (i.e., Bowditch phenomenon), which could help the immature heart tolerate increased afterload, has remained controversial. Using a piglet model, Schmidt et al. demonstrated that the force-frequency relationship exists in the neonate (12), whereas, Wiegerinck et al. showed a flat force-frequency relationship in human tissue obtained from neonatal hearts with structural malformations (4). This latter study, however, demonstrated a significant increase in frequency dependent acceleration of relaxation in the neonatal group, a finding which could support the presence of tolerance to tachycardia and corroborate the findings from our previous *in vivo* animal investigation.

In the present study, we sought to explore how varying preload and afterload impacts tolerance to tachycardia in an *in vivo* swine model at two different but very close post-natal developmental stages. We hypothesized that post-natal maturation would be associated with better response to increase in preload and afterload when challenged by concomitant tachycardia.

## Materials and Methods

We report three series of experimentations. Six groups of 7 Landrace cross piglets were included (total of 42 piglets, both males and females used). Three groups of neonatal (NP, NPV, NPA; respectively: no loading modification, hypervolemia and increase afterload; 1–3 days of age) and 3 groups of young (YP, YPV, YPA; respectively: no loading modification, hypervolemia and increase afterload; 14–17 days of age) piglets were subjected to the pacing protocol. These age groups were chosen because the heart rate is relatively stable in the first 2 weeks of life (13) and they have been previously used to describe early cardiovascular maturational changes (13, 14). This study was approved of by the animal research ethics board at the University of Alberta and was designed in accordance with the Canadian Council on Animal Care as well as the European Convention for the Protection of Vertebrate Animals used for Experimental and other Scientific Purposes (Council of Europe No 123, Strasbourg 1985) guidelines. Experiments were completed in 2016. Partial data from two of the groups (NP, YP) has been previously published (6). With the exception of Table 1, which shows that the groups were comparable at baseline, no direct statistical comparisons were made between subjects from the 3 series of experiments (see statistical analysis section for details).

**Table 1:** Group comparisons before intervention and the tachycardia protocol.

	**NP**	**NPV**	**NPA**	**ANOVA p**	**YP**	**YPV**	**YPA**	**ANOVA p**
Heart rate (beats min^−1^)	142 ± 8	148 ± 7	143 ± 4	0.82	159 ± 5	147 ± 7	169 ± 4	0.07
LVO (ml kg^−1^ min^−1^)	245 ± 16	271 ± 11	243 ± 23	0.46	257 ± 13	231 ± 17	263 ± 20	0.40
Mean BP (mmHg)	45 ± 2	66 ± 5^a^	59 ± 2^a^	0.001	59 ± 4	68 ± 12	60 ± 4	0.30
dP/dt (mmHg s^−1^)	1,574 ± 182	2,066 ± 151	2,253 ± 234	0.06	1,738 ± 148	1,907 ± 167	2,110 ± 166	0.29
Negative dP/dt (mmHg s^−1^)	1,598 ± 83	2,480 ± 243^a^	2,136 ± 140^a^	0.006	2,470 ± 226	2,570 ± 138	2,394 ± 185	0.80
LVEDP (mmHg)	8.39 ± 2.22	9.06 ± 2.15	8.38 ± 2.19	0.97	9.04 ± 0.84	9.53 ± 2.61	10.03 ± 1.07	0.92
Tau (ms)	23.8 ± 2.1	20.0 ± 1.2	22.4 ± 2.0	0.37	22.0 ± 2.3	18.5 ± 1.1	18.9 ± 0.7	0.23
CVP (cmH_2_O)	2.83 ± 0.77	2.29 ± 0.33	5.42 ± 1.01	0.02	3.61 ± 0.77	4.06 ± 0.87^b^	7.4 ± 1.0^a^^,^ ^b^	0.01

NP, neonatal piglets; NPV, neonatal piglets allocated to the hypervolemia group; NPA, neonatal piglets allocated to the increase afterload group; YP, young piglets; YPV, young piglets allocated to the hypervolemia group; YPA, young piglets allocated to the increase afterload group; LVO, left ventricular output; BP, blood pressure; LVEDP, left ventricular end diastolic pressure; CVP, central venous pressure.

a Higher than the NP or YP group (p < 0.05).

b *Significant difference between the hypervolemia group and the increase afterload group (p < 0.05)*.

### Anesthesia and Instrumentation

Piglets were placed under Isoflurane 2–5% in nitrous oxide (5 l·min^−1^) and oxygen (5 l·min^−1^). Tracheotomy and external jugular venous access were performed through a neck cut-down to allow for mechanical ventilation (Ohio 30/70 Proportioner Anesthesia machine) and administration of medication. Low dose propofol (85 mcg·kg^−1^·min^−1^) along with a D10W solution (5 ml/kg/h) was given and the desired level of anesthesia was adjusted with isoflurane (0.5–2%). The lowest concentration of isoflurane was used in order to achieve quiet sleep with some respiratory efforts. Vascular sheaths (5–6.5 Fr) were introduced in the carotids and the right external jugular vein for catheter placement. A fluid filled catheter (3.5 Fr) was positioned in the superior vena cava for central venous pressure monitoring. Arterial blood pressure was monitored through a side port on one of the carotid sheath. A 4 Fr pacemaker lead was introduced into the right atrial appendage and connected to an external pacemaker (Model 5,388, Medtronic, Dublin, Ireland). A high-fidelity pressure catheter (3.5 Fr for NPs and 5 Fr for YPs; SPR-524 and SPR-350S, Millar Instruments, Houston, Texas) was positioned in the LV. Thus, all the invasive monitoring devices were introduced by intravascular means through the neck cutdown.

For the increase afterload groups (NPA and YPA), a coronary angioplasty balloon catheter or a pediatric valvuloplasty balloon catheter was introduced through one of the carotid sheath and positioned at the level of the diaphragm in the descending aorta. Balloon size (3–6 mm in diameter) was selected to produce a subtotal obstruction of the aorta confirmed by echocardiography (minimal but present visible flow by color Doppler, evidence of aortic obstruction by Doppler both at and distal to the obstruction) (Vivid 7 ultrasound machine, GE Healthcare, Little Chalfont, United Kingdom). The balloon was then deflated in order to proceed to baseline measurements. Catheter placement was done under both fluoroscopic and echocardiographic guidance. Blood gas analysis was performed before and immediately after the pacing protocol (iStat system; Abbott Point of Care, Princeton, New Jersey). Rectal temperature and pulse oximetry were continuously monitored during the experiments. Piglets were euthanized with an overdose of pentobarbital (100 mg·kg^−1^ iv) at the end of the experiments.

### Baseline Measurements and the Pacing Protocol

After a 30-min recovery period, baseline parameters were recorded. The hypervolemia piglets (NPV and YPV) then received a 60 ml/kg warm normal saline bolus over 20 min. In the increase afterload piglet groups (NPA and YPA), the balloon was inflated to produce a sub-total aortic occlusion with demonstration of continuous holodiastolic flow and reduced systolic peak in the abdominal aorta tracing, a typical feature of aortic coarctation. The hypervolemia and increase afterload groups were then given 15 min of stabilization before post-intervention baseline values were collected. Atrial pacing was then started at 200 beats/min and increased by 10 beats·min^−1^ increments until 300 beats·min^−1^ as it had previously been shown to induce heart failure in the neonatal piglet heart (15). A 30 s stabilization period occurred before recordings were made at each of the heart rate.

### Recorded Parameters

Left ventricular output was calculated using the following formula: LVO = (TVI·CSA·HR)/weight, where TVI is the time velocity integral obtained by placing the pulse wave Doppler sample at the level of the aortic valve from an apical 5-chamber view, averaged over 7–10 cycles, CSA is the cross-sectional area of the aortic valve annulus measured from the parasternal long axis (calculated as CSA = π^*^radius^2^), HR is heart rate in beats per minute (beat/min), and weight of the animal in kilograms (kg). Five none consecutive values out of 532 had to be averaged from the heart rate over and under the missing value due to failure to record.

Data from the pressure catheters were displayed and recorded continuously using Ponemah software (Data Science International, St. Paul, Minnesota). Blood pressure, dP/dt, negative dP/dt, and tau were averaged from 20 to 25 heart cycles. Tau was calculated using the following formula:

$$\begin{array}{l} \tau =\;-\;\frac{N\;\sum x^{2}-\;\sum x\;*\;\sum x}{N\;\sum \left[x\;*\;\ln\left(p\right)\right]-\;\sum x\;*\;\sum \ln\left(p\right)}\; \end{array}$$

where *N* is the number of points used in the calculation, *x* is the delta time in seconds at each sampled point (starting from the minimum dP/d*t* point), and *p* is the LV pressure value at each sampled point. LVEDP was manually averaged from 7 to 10 heart beats to avoid where possible intrathoracic pressure variations related to mechanical ventilation. CVP was averaged from five cardiac cycles.

### Statistical Analysis

Baseline parameters were compared between groups using ANOVA. Aside from baseline, subjects from the NP and YP groups were not used for statistical comparisons to the other groups. Effects of volume loading and increasing afterload (i.e., intervention) within group was assessed with paired *t-*test. Within group variation from post-intervention value to 300 bpm was assessed using repeated measure ANOVA with Greenhouse-Geisser adjustment. If significant, the post-intervention value could be compared with the value at 300 bpm and the peak value with both the post-intervention and the 300 bpm values. Holm-Bonferroni adjustment was used for multiple comparisons. Data is presented as mean ± SEM unless specified. Were appropriate, equivalent non-parametric tests were used.

## Results

### Baseline (Before Altering Cardiac Loading Condition)

Neonatal groups (NP, NPV, NPA) had similar weight (*p* = 0.37) as did the young piglet groups (YP, YPV, YPA) (*p* = 0.33). As expected, the young piglet groups were heavier than the neonatal piglets (5.56 ± 0.19 vs. 1.83 ± 0.05 kg, *p* < 0.001). All 3 neonatal groups had similar baseline left ventricular output (LVO) (245 ± 16, 271 ± 11, and 243 ± 23 ml·kg^−1^·min^−1^, for NP, NPV, and NPA, respectively, *p* = 0.46) as did the 3 young infant groups (257 ± 13, 231 ± 17, and 263 ± 20 ml·kg^−1^·min^−1^, for YP, YPV, and YPA, respectively, *p* = 0.40). Taken together, at baseline (before intervention) the neonatal groups had a similar LVO when compared to the young piglet groups (253 ± 10 vs. 251 ± 10 ml·kg^−1^·min^−1^, *p* = 0.87). Baseline comparisons between groups are shown in Table 1. Within the neonatal groups, the NP group had a lower baseline mean blood pressure (*p* < 0.05). Negative dP/dt, a load dependent parameter, was also lower for NP (*p* < 0.05).

### Effect of Increased Preload

Prior to atrial pacing, with increased preload, both NPV and YPV increased their LVO (Table 2). Although the neonatal group tended to demonstrate a greater increase in LVO in response, the difference did not reach statistical significance (+23 ± 5% vs. +14 ± 4%, *p* = 0.23). Both age groups demonstrated a trend toward an increase in blood pressure, contractility (i.e., dP/dt), left ventricular diastolic pressure (LVEDP) and central venous pressure (CVP).

**Table 2:** Effect of increasing preload.

	**Baseline**	**Post volume administration**	***p***
Neonatal piglets (NPV)			
LVO (ml·kg^−1^·min^−1^)	271 ± 11	334 ± 23	0.007
Heart rate (beats·min^−1^)	148 ± 7	161 ± 7	0.06
Mean blood pressure (mmHg)	66 ± 5	71 ± 4	0.19
dP/dt (mmHg·s^−1^)	2,066 ± 151	2,381 ± 112	0.05
Negative dPdt (mmHg·s^−1^)	2,480 ± 243	2,447 ± 167	0.86
LVEDP (mmHg)	9.1 ± 2.2	12.0 ± 2.1	0.03
Tau (ms)	20.0 ± 1.2	20.4 ± 2.0	0.80
CVP (cmH_2_O)	2.29 ± 0.33	3.41 ± 0.54	0.01
Young infant piglets (YPV)			
LVO (ml·kg^−1^·min^−1^)	231 ± 17	263 ± 21	0.03
Heart rate (beats·min^−1^)	147 ± 7	155 ± 5	0.12
Mean blood pressure (mmHg)	68 ± 4	78 ± 4	0.03
dP/dt (mmHg·s^−1^)	1,907 ± 167	2,377 ± 141	0.04
Negative dPdt (mmHg·s^−1^)	2,570 ± 138	3,097 ± 216	0.07
LVEDP (mmHg)	9.5 ± 2.6	15.4 ± 1.6	0.01
Tau (ms)	18.5 ± 1.1	20.7 ± 1.1	0.03
CVP (cmH_2_O)	4.07 ± 0.86	7.04 ± 0.77	0.002

*NPV, neonatal piglet in the hypervolemia group; YPV, young piglets in the hypervolemia group; LVO, left ventricular output; LVEDP, left ventricular end diastolic pressure; CVP, central venous pressure*.

### Effect of Increased Afterload

When reported as a ratio of the balloon over aortic valve annulus diameter, balloons selected for aortic obstruction had a similar size for both NPA and YPA groups (65 ± 3 vs. 63 ± 1%, *p* = 0.47, respectively). Both groups increased their blood pressure proximal to the obstruction (Table 3 presents data after stabilization). Increasing afterload did not affect LVO nor heart rate in either group before the pacing protocol.

**Table 3:** Effect of increasing afterload.

	**Baseline**	**Increased afterload**	***P***
Neonatal piglets (NPA)			
LVO (ml·kg^−1^·min^−1^)	243 ± 23	249 ± 26	0.34
Heart rate (beats·min^−1^)	143 ± 5	148 ± 4	0.12
Mean blood pressure (mmHg)	59 ± 2	68 ± 3	0.02
dP/dt (mmHg·s^−1^)	2,253 ± 234	2,678 ± 195	0.002
Negative dPdt (mmHg·s^−1^)	2,136 ± 140	2,376 ± 152	0.044
LVEDP (mmHg)	8.38 ± 2.19	8.49 ± 2.08	0.812
Tau (ms)	22.39 ± 2.01	22.92 ± 1.88	0.559
CVP (cmH_2_O)	5.42 ± 1.01	5.34 ± 0.95	0.866
Young infant piglets (YPA)			
LVO (ml·kg^−1^·min^−1^)	263 ± 20	255 ± 20	0.16
Heart rate (beats·min^−1^)	169 ± 6	171 ±8	0.62
Mean blood pressure (mmHg)	60 ± 4	82 ± 7	0.008
dP/dt (mmHg·s^−1^)	2,110 ± 166	2,384 ± 173	0.116
Negative dPdt (mmHg·s^−1^)	2,394 ± 185	2,815 ± 79	0.039
LVEDP (mmHg)	10.03 ± 1.07	11.15 ± 1.24	0.222
Tau (ms)	18.88 ± 0.71	23.27 ± 1.04	0.011
CVP (cmH_2_O)	7.40 ± 0.96	8.17 ± 0.96	0.014

*NPA, neonatal piglets in the increase afterload group; YPA, young piglets in the increase afterload group; LVO, left ventricular output; LVEDP, left ventricular end diastolic pressure; CVP, central venous pressure*.

### Tolerance to the Pacing Protocol

**Figure 1:**
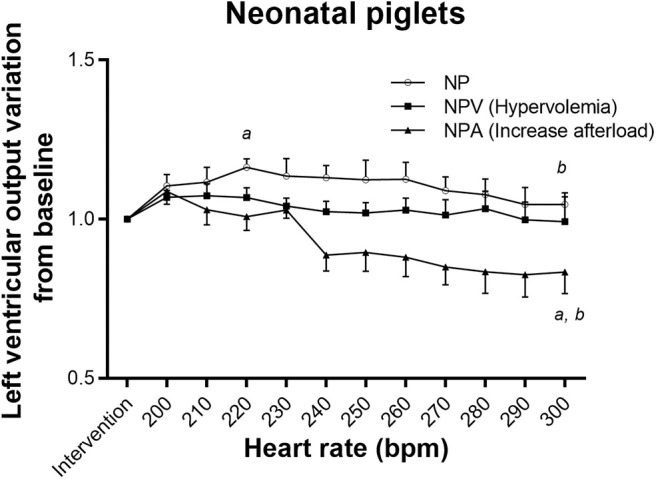
Tolerance to tachycardia in neonatal groups. In NP and NPA (increase afterload) groups, left ventricular output varied with pacing. It slightly increased with pacing for NP. Left ventricular output decreased significantly at faster heart rates in NPA. In group NPV (hypervolemia), pacing had no effect on LVO. ^a^Different from post-intervention value (*p* < 0.05). ^b^Different from maximum value reached (*p* < 0.05).

**Figure 2:**
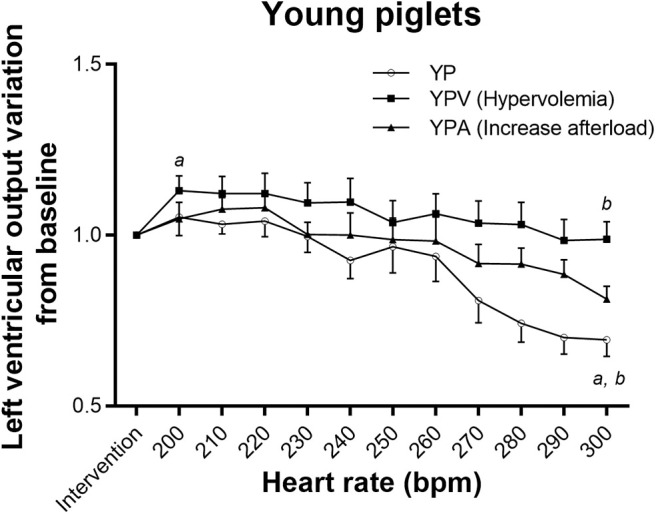
Tolerance to tachycardia in young infant groups. Impact of atrial pacing on left ventricular output in the three groups. Both YP and YPA (increase afterload) had a decreased output with pacing (adjusted repeated measure ANOVA *p*-value < 0.001 and 0.001, respectively). However, the paired comparison between the left ventricular output after intervention and at 300 bpm became non-significant in the YPA group after adjustment for multiple comparisons (*p*-value increased from 0.04 to 0.09) due to small numbers. Left ventricular output increased in YPV (hypervolemia) before going back initial values. ^a^Different from post-intervention value (*p* < 0.05). ^b^Different from maximum value reached (*p* < 0.05).

The NP group tolerated tachycardia very well, maintaining baseline LVO even at 300 bpm (Figure 1), but YP did not (repeated measure ANOVA *p* < 0.001), decreasing rapidly from 230 bpm (Figure 2). This lack of tolerance to fast heart rates in the young infant piglet group was completely corrected with increasing preload before submitting the animals to pacing (YPV). Reduction in LVO during tachycardia was not exaggerated by increased afterload. In comparison, NPV maintained their output through pacing (*p* = 0.27), but with increased afterload, NPA did not (*p* = 0.005, lower than baseline at 240 bpm and at higher heart rates). Raw data, not normalized to post-intervention values, is available as supplemental material (Figures S1, S2). Blood gases acquired prior to and during the pacing protocol were all within an acceptable range (pH between 7.21 and 7.41, see Table S1).

**Figure 3:**
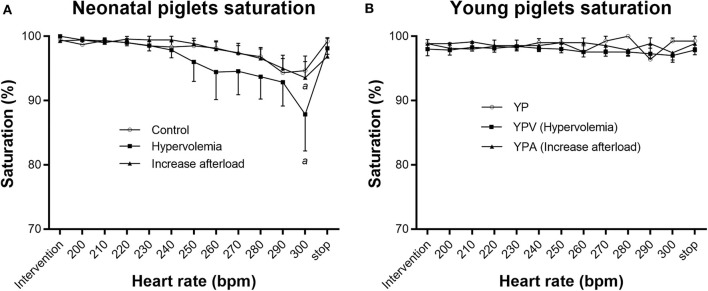
Saturation through pacing protocol. Pacing affected oxygen saturation levels in NPV (hypervolemia) and NPA (increase afterload) groups (Friedman test *p* < 0.001 for both) **(A)**. Pacing did not affect saturation in the YP groups **(B)**. Paired comparisons based on Wilcoxon Signed Rank Sum test (*p* < 0.05). ^a^Different from before pacing protocol value (*p* < 0.05).

Although NPV maintained their LVO similarly to NP, mean oxygen saturation decreased at 300 bpm (before pacing 99.7 ± 0.3% vs. 300 bpm 87.9 ± 5.7%, wilcoxon signed rank test *p* = 0.04) (Figure 3A). Four out of 7 piglets in this group presented with clinically relevant desaturation (i.e., <95%) associated with a right to left shunt at the patent foramen ovale documented by echocardiography. Importantly, there was no difference in the LVO between NPV with and without right to left atrial shunting (−15 ± 40 ml·kg^−1^·min^−1^ vs. +6 ± 29 ml·kg^−1^·min^−1^, respectively, *p* = 0.79). Similar desaturations at fast heart rate were also seen in 3 piglets from the NPA group. Immediate recovery of desaturation was observed on cessation of pacing for all subjects. Young infant piglet groups did not demonstrate desaturation (Friedman *p*-values of 0.98, 0.45, and 0.86, for YP, YPV, and YPA, respectively) (Figure 3B).

Blood pressure differences between NP and the NPV and NPA groups observed before the tachycardia protocol were similar from 260 bpm to 300 bpm. All other invasive parameters through pacing are available as supplemental material (Table S2). LVEDP, tau and CVP demonstrated a U-shape pattern in all six groups (repeated measure ANOVA quadratic *p*-value for all the animals of < 0.001 for all three variables). The nadir for CVP was reached early in the neonatal piglet groups (all three at 210 bpm) in comparison to the young piglet groups (280, 240, and 240 bpm, for YP, YPV, and YPA, respectively).

## Discussion

The current work contributes further to our understanding of how changes in loading impact the immature heart's tolerance of atrial tachycardia. It also advances our understanding of how early maturation potentially influences myocardial reserve. We found neonatal hearts to tolerate tachycardia at baseline and with increased preload, whereas increased afterload was poorly tolerated with tachycardia. In contrast, slightly more mature hearts did not tolerate tachycardia but tolerance improved with augmented preload, and increased afterload had no added impact on output during tachycardia.

Our finding of increased LVO with low grade tachycardia in the NP group is in accordance with early fetal studies (16) but is in contrast to a single neonatal study that employed a lamb model (17), using ventricular instead of atrial pacing. Aside from the slightly different age groups used in this latter study, the decision to pace the ventricle could have negated any benefit of tachycardia with loss of the contribution of atrial contraction as it has been previously shown to importantly contribute to ventricular filling and maintenance of the cardiac output in the newborn (18). We found increased preload to augment the neonatal output at baseline as has been previously published (8–10) in animal studies, but we also demonstrated that the output was maintained even with atrial tachycardia. More importantly, increasing ventricular preload through volume infusion before atrial tachycardia allowed the YPV piglets to maintain their cardiac output, which was not the case in the YP group.

In late fetal life, a larger proportion of the cardiac output is assumed by the right ventricle (19) and the LV faces low afterload due in the presence of the placental circulation. Fetal ventricular growth is achieved through hyperplasia. After birth, removal of the placental circulation induces a rapid disproportional growth of the LV (20, 21) through myocyte hypertrophy in order to sustain this increase afterload (22) that normalize wall stress (23). The level of myocardial “contractility reserve” in response to further increase in afterload in the immediate neonatal period has therefore been questioned (24). We did not show a decrease in LVO after increasing afterload in the absence of atrial tachycardia in our NP group. However, their poor tolerance to increase afterload became manifested with atrial tachycardia. Postnatal maturation indeed seemed to slightly increase tolerance to afterload as reduction in cardiac output during tachycardia was not exaggerated by increased afterload. One could argue that a weaker force frequency relationship due to high early neonatal expression of the Na-Ca exchanger resulting in lower sarcoplasmic reticulum Ca loading might contribute to this finding (4). However, in this study, both NPA, and YPA demonstrated a similar non-significant increase of dP/dt with pacing. Also, we hypothesize that the rapid LV hypertrophy might impact compliance through intrinsic muscle stiffness (23), requiring adequate filling to maintain output, thus explaining our finding of better tolerance to tachycardia with high preload in the YP group. Many other aspects of myocardial maturation could, however, be involved, including but not likely limited to the rapid evolution of cardiac metabolism within a few days after birth (5) (i.e., decrease in glycolytic rates and increase fatty acid beta-oxidation). More comprehensive integration of knowledge gained from fundamental studies with *in vivo* testing through the use of animal models with an intact cardiovascular system such as this one are required to see the global effects of maturational processes.

A surprising finding of this study was the presence of oxygen desaturation in the neonatal piglets which was amplified by volume loading, and associated with right to left atrial shunting documented by echocardiography. As LVEDP, CVP, and tau showed a U-shape pattern for all 6 groups of piglets, we are confident that levels of tachycardia approached early stages of biventricular heart failure. The fact that only neonatal piglets demonstrated significant desaturation could imply the right ventricle of the immature heart has lower tolerance to tachycardia when compared to young piglets. An alternative explanation could be that the foramen ovale was no longer patent in the YPs; however, anatomic closure was not confirmed at the time of necropsy. The impact of tachycardia on right ventricular function in the neonatal period should be explored further. Finally, LV failure could have resulted in pulmonary edema and subsequent desaturation but immediate return to normal saturation following the cessation of pacing also argues against this possibility. One could also argue that better tolerance to tachycardia in the NP and NPA group could have been secondary to an increase LV preload from a right to left shunt at atrial level. However, piglets with significant desaturation in the NPA group had a trend toward lower output than the ones without desaturation, arguing against this hypothesis.

### Clinical Relevance

Our results suggest an initial approach based on limiting afterload in the early neonatal heart and maximizing preload in the young infant heart could maximize cardiac output when tachycardia is present. Fear of poor tolerance to increase preload or tachycardia in the neonate, based on echocardiographic LV measurements obtained at rest (1), might not be warranted. Furthermore, we believe global cardiovascular perinatal transition should not be oversimplified, being viewed as a linear process, from worst to better. Randomized control trials to test different approaches to shock in the early life are needed.

### Limitation

The main limitation of this study was the use of electrical pacing to induce tachycardia. Naturally occurring tachycardia in the context of an acute illness in the newborn involves many other physiologic processes that can impact tolerance to the tachycardia, including but not limited to adrenergic stimulation and microcirculation dysfunction (25). Although small differences between groups at baseline were to be expected when using a small number of animals, one difference between our groups at baseline requires discussion. The NP group had lower blood pressure at baseline. One could argue that a lower afterload could have facilitated tolerance to tachycardia in that group. However, since this difference disappeared at higher heart rates, we do not believe this affected the results observed at higher heart rates between groups. Although blood pH was normal in both the NPA and the YPA groups before pacing, base excess was lower than in the other conditions following the increase in afterload (Table S1). While it is possible ventricular function could have been minimally affected, given that the pH and base excess were similar at the beginning and the end of the tachycardia protocol, we believe the hemodynamic stress between the NPA and YPA groups to have been very similar.

As an *in vivo* model was chosen to study the impacts of loading conditions and tachycardia, one must acknowledge that the findings are the results of complex interactions that are not comparable to an *in vitro* Langendorff type experiment. For example, increasing afterload in the YPA group also slightly increased CVP, while inducing tachycardia resulted in a significant decrease in CVP in the YPY group. The resulting LVO is thus the result of multiple uncontrolled hemodynamic adaptative and perhaps maladaptive mechanisms. The first days of the perinatal period are also marked by contraction of extracellular volume and insensible fluid loss (26) as well as rapid decline in pulmonary vascular resistance (27). This could have affected the homogeneity of the response to hemodynamic stress in the neonatal groups (aged 1–3 days). Although formal subgroup statistical analysis was not possible due to lack of power, individual response to tachycardia within these 3 groups were qualitatively very similar. Our choice of an *in vivo* model was deliberate, despite its complexity, as we sought to reproduce clinical scenarios. We believe this study is thus complementary to *in vitro* studies.

## Conclusions

Birth involves rapid changes in the cardiovascular system. We have shown that even within a short window of early maturation there are important changes that occur in myocardial tolerance of atrial tachycardia, suggesting acute management of the critically ill neonate should integrate such concepts. Our results advocate for an initial approach based on limiting afterload in the early neonatal heart and maximizing preload in the young infant heart could maximize cardiac output when atrial tachycardia is present.

## Data Availability Statement

All datasets presented in this study are included in the article/Supplementary Material.

## Ethics Statement

The animal study was reviewed and approved by Animal research ethics board at the University of Alberta.

## Author Contributions

EF-P, NK, and LH: original idea. EF-P, NK, JC, LM, P-YC, and LH: experimentations, data analysis, and manuscript. All authors contributed to the article and approved the submitted version.

## Conflict of Interest

The authors declare that the research was conducted in the absence of any commercial or financial relationships that could be construed as a potential conflict of interest.
