# Supplementary material for: Effects of Early Myocardial Postnatal Maturation on Tolerance to Atrial Tachycardia With Altered Loading Conditions: An in vivo Swine Model
Source: Front Pediatr. 2020 Jun 25;8:346. doi: 10.3389/fped.2020.00346 (PMC7330128; doi:10.3389/fped.2020.00346)
Supplement: Supplementary file 1 [file Data_Sheet_1.docx]

# Supplemental material

**Table S1 Blood gas**

| NP | Before pacing | After pacing | t-test p |
| --- | --- | --- | --- |
| pH | 7.40 ± 0.03 | 7.36 ± 0.06 | 0.3 |
| pCO2 (torr) | 35.4 ± 2.5 | 38.4 ± 5.5 | 0.5 |
| Excess base (mEq·L^-1^) | -2.57 ± 1.59 | -5.14 ± 1.47 | 0.2 |
|  |  |  |  |
| NPV (volume load) |  |  |  |
| pH | 7.41 ± 0.05 | 7.35 ± 0.03 | 0.07 |
| pCO2 (torr) | 38.0 ± 2.7 | 38.3 ± 3.6 | 0.9 |
| Excess base (mEq·L^-1^) | -1.50 ± 1.67 | -4.33 ± 1.69 | 0.2 |
|  |  |  |  |
| NPA (increase afterload) |  |  |  |
| pH | 7.36 ± 0.04 | 7.23 ± 0.04 | 0.04 |
| pCO2 (torr) | 33.1 ± 2.7 | 37.3 ± 4.7 | 0.4 |
| Excess base (mEq·L^-1^) | -6.86 ± 2.21 | -12.14 ± 1.53 | 0.06 |
|  |  |  |  |
| YP |  |  |  |
| pH | 7.37 ± 0.03 | 7.26 ± 0.03 | 0.06 |
| pCO2 (torr) | 39.6 ± 1.8 | 42.8 ± 3.4 | 0.5 |
| Excess base (mEq·L^-1^) | -2.43 ± 0.95 | -8.29 ± 0.92 | 0.001 |
|  |  |  |  |
| YPV (volume load) |  |  |  |
| pH | 7.42 ± 0.03 | 7.38 ± 0.03 | 0.2 |
| pCO2 (torr) | 33.8 ± 0.7 | 36.2 ± 2.2 | 0.3 |
| Excess base (mEq·L^-1^) | -2.14 ± 2.0 | -4.0 ± 0.7 | 0.3 |
|  |  |  |  |
| YPA (increase afterload) |  |  |  |
| pH | 7.34 ± 0.03 | 7.21 ± 0.04 | 0.03 |
| pCO2 (torr) | 33.9 ± 2.8 | 44.2 ± 4.2 | 0.03 |
| Excess base (mEq·L^-1^) | -7.71 ± 0.84 | -10.43 ± 1.45 | 0.2 |

**Table S2 Effect of pacing**

The p value is from the adjusted repeated measure ANOVA analysis.

*^a^* Different from ‘before tachycardia’ value (p < 0.05, adjusted for multiple comparisons)

*^b^* Different from maximum or minimum value (p < 0.05, adjusted for multiple comparisons)

|  | Groups | Before tachycardia (after intervention on loading condition) | Maximum or minimum value if outside the ‘before tachycardia’ to 300bpm value interval | Value at 300bpm | ANOVA p |
| --- | --- | --- | --- | --- | --- |
| Mean BP (mmHg) | NP | 45 ± 2 | 51 ± 3 | 48 ± 4 | 0.29 |
|  | NPV | 71 ± 4 | 73 ± 5 | 55 ± 7*^b^* | 0.04 |
|  | NPA | 68 ± 3 | 72 ± 3*^a^* | 59 ± 3*^a,b^* | < 0.001 |
|  | YP | 59 ± 4 | - | 44 ± 5 | 0.05 |
|  | YPV | 78 ± 4 | - | 68 ± 1*^a^* | 0.006 |
|  | YPA | 82 ± 7 | 85 ± 10 | 81 ± 11 | 0.53 |
| dP/dt (mmHg·s^-1^) | NP | 1574 ± 182 | 2497 ± 146 | 2051 ± 199 | 0.05 |
|  | NPV | 2381 ± 112 | 2692 ± 154 | 2236 ± 326 | 0.26 |
|  | NPA | 2678 ± 194 | 2918 ± 235 | 2523 ± 262 | 0.26 |
|  | YP | 1738 ± 148 | 2501 ± 169 | 1967 ± 184 | 0.05 |
|  | YPV | 2377 ± 141 | - | 2167 ± 74 | 0.08 |
|  | YPA | 2384 ± 173 | 2844 ± 393 | 2764 ± 335 | 0.27 |
| Negative dP/dt (mmHg·s^-1^) | NP | 1598 ± 83 | 2202 ± 139*^a^* | 2051 ± 199 | 0.015 |
|  | NPV | 2447 ± 167 | 2677 ± 260 | 2305 ± 377 | 0.50 |
|  | NPA | 2376 ± 152 | 2715 ± 166 | 2207 ± 187*^b^* | 0.016 |
|  | YP | 2470 ± 226 | - | 1838 ± 194 | 0.18 |
|  | YPV | 3097 ± 216 | - | 2736 ± 134*^a^* | 0.01 |
|  | YPA | 2815 ± 79 | 3029 ± 410 | 2828 ± 347 | 0.60 |
| LVEDP (mmHg) | NP | 8.4 ± 2.2 | 5.7 ± 0.5 | 6.5 ± 0.7 | 0.49 |
|  | NPV | 12.0 ± 2.1 | 3.5 ± 0.7*^a^* | 3.8 ± 0.5*^a^* | 0.001 |
|  | NPA | 8.5 ± 2.1 | 3.8 ± 1.3*^a^* | 4.8 ± 1.1 | 0.038 |
|  | YP | 9.0 ± 0.8 | 4.6 ± 0.9*^a^* | 5.2 ± 0.7*^a^* | 0.004 |
|  | YPV | 15.4 ± 1.6 | 3.5 ± 1.0*^a^* | 4.0 ± 0.9*^a,b^* | < 0.001 |
|  | YPA | 11.1 ± 1.2 | 7.6 ± 1.2*^a^* | 8.4 ± 1.2 | 0.026 |
| Tau (ms) | NP | 23.8 ± 2.1 | 16.5 ± 1.8*^a^* | 20.4 ± 2.1*^b^* | 0.039 |
|  | NPV | 20.4 ± 2.0 | 15.5 ± 1.1 | 18.4 ± 1.6 | 0.25 |
|  | NPA | 22.9 ± 1.9 | 18.6 ± 1.5 | 20.5 ± 1.8 | 0.26 |
|  | YP | 22.0 ± 2.3 | 18.3 ± 1.9 | 19.7 ± 1.4 | 0.52 |
|  | YPV | 20.7 ± 1.1 | 18.0 ± 0.8 | 18.8 ± 0.7 | 0.24 |
|  | YPA | 23.3 ± 1.0 | 22.0 ± 1.2 | 22.1 ± 0.7 | 0.64 |
| CVP (cmH_2_O) | NP | 2.8 ± 0.8 | 1.7 ± 0.4 | 3.3 ± 0.6 | 0.05 |
|  | NPV | 3.4 ± 0.5 | 1.8 ± 0.2 | 2.8 ± 0.4 | 0.06 |
|  | NPA | 5.3 ± 0.9 | 4.31 ± 0.8 | 5.9 ± 0.7 | 0.072 |
|  | YP | 3.6 ± 0.8 | 1.0 ± 0.7 | 1.9 ± 0.4 | 0.10 |
|  | YPV | 7.0 ± 0.8 | 3.61 ± 0.8*^a^* | 5.5 ± 0.5*^a,b^* | 0.001 |
|  | YPA | 8.2 ± 1.0 | 7.0 ± 0.5 | 8.3 ± 0.3 | 0.25 |

**
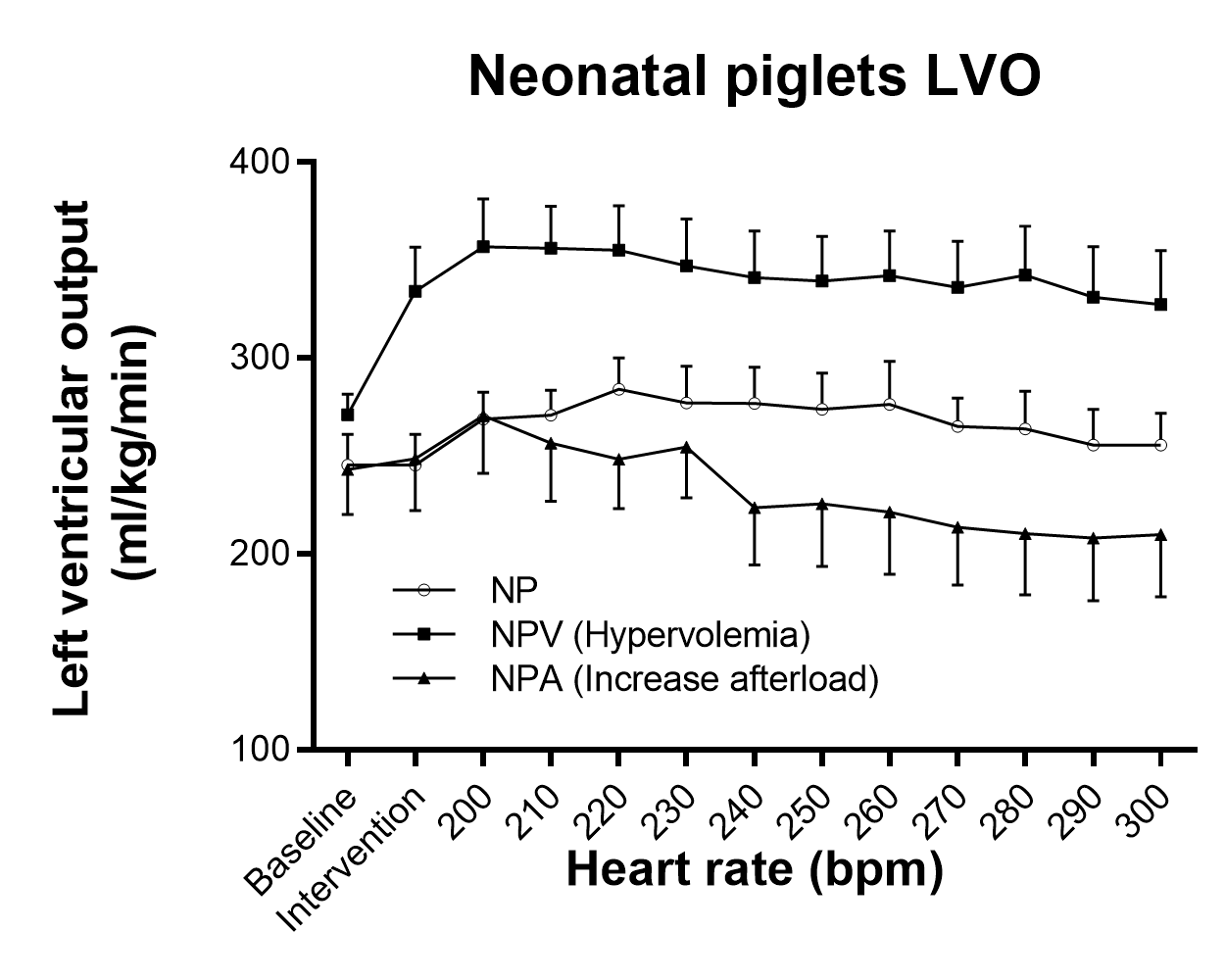
**

**Figure S1 NP groups LVO in ml/kg/min from baseline to 300bpm (no statistical analysis)**

**
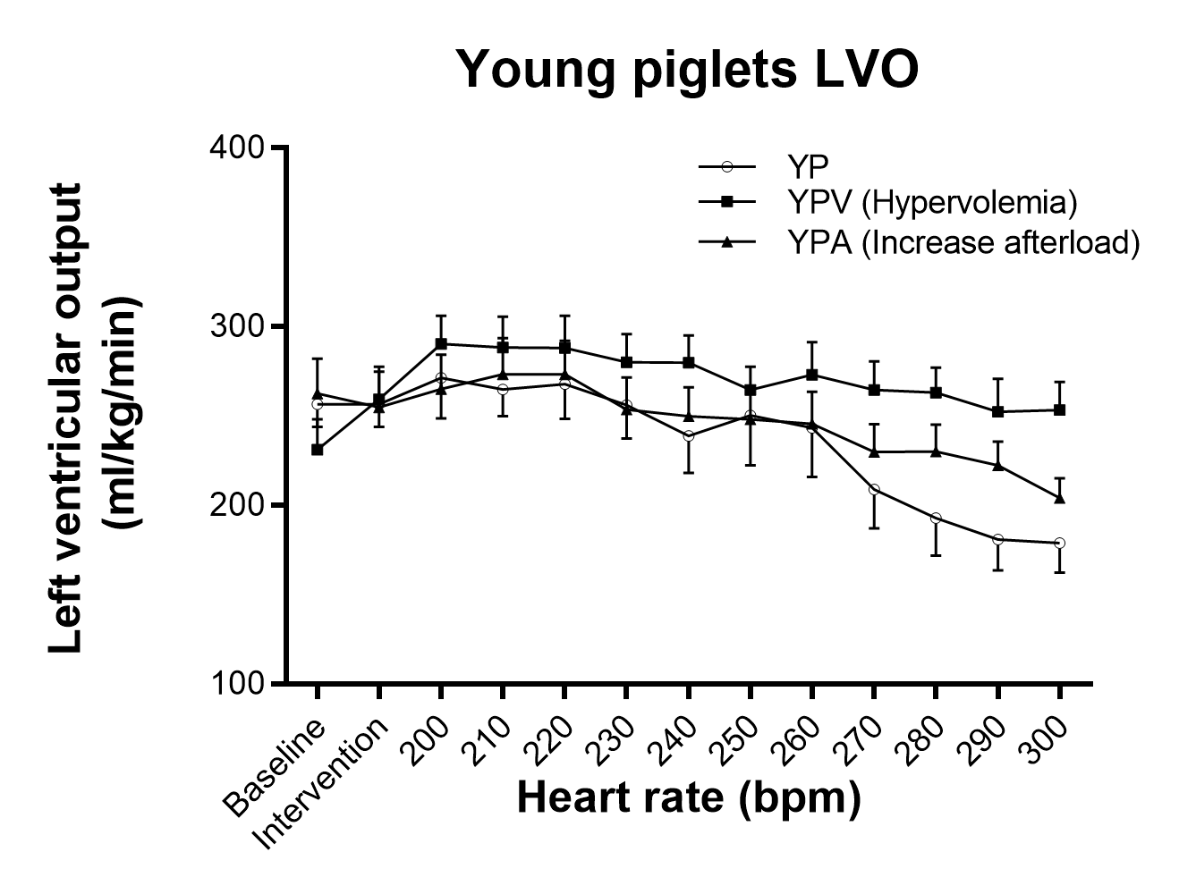
**

**Figure S2 YP groups LVO in ml/kg/min from baseline to 300bpm (no statistical analysis)**
